# The Antidepressant-like Effect of Flavonoids from *Trigonella Foenum-Graecum* Seeds in Chronic Restraint Stress Mice via Modulation of Monoamine Regulatory Pathways

**DOI:** 10.3390/molecules24061105

**Published:** 2019-03-20

**Authors:** Jiancheng Wang, Cuilin Cheng, Chao Xin, Zhenyu Wang

**Affiliations:** Harbin Institute of Technology, 92 West Dazhi Street, Nangang District, Harbin 150001, China; wangjiancheng3612@126.com (J.W.); wjc2386026898@163.com (C.X.)

**Keywords:** flavonoids, depression, monoamine neurotransmitters, monoamine oxidase A, KLF11, SIRT1

## Abstract

Fenugreek (*Trigonella Foenum-Graecum*) seeds flavonoids (FSF) have diverse biological activities, while the antidepressant-like effect of FSF has been seldom explored. The aim of this study was to evaluate the antidepressant-like effect of FSF and to identify the potential molecular mechanisms. LC-MS/MS was used for the determination of FSF. Chronic restraint stress (CRS) was used to establish the animal model of depression. Observation of exploratory behavior in the forced swimming test (FST), tail suspension test (TST) and sucrose preference test (SPT) indicated the stress level. The serum corticosterone (CORT) level was measured. The monoamine neurotransmitters (5-HT, NE and DA) and their metabolites, as well as monoamine oxidase A (MAO-A) enzyme activity in the prefrontal cortex, hippocampus and striatum, were evaluated. The protein expression levels of KLF11, SIRT1, MAO-A were also determined by western blot analysis. The results showed that FSF treatment significantly reversed the CRS-induced behavioral abnormalities, including reduced sucrose preference and increased immobility time. FSF administration markedly restored CRS induced changes in concentrations of serum corticosterone, prefrontal cortex neurotransmitters (NE, 5-HT and DA), hippocampus neurotransmitters (NE, 5-HT and DA) and striatum neurotransmitters (NE). FSF treatment exhibited significant inhibition of MAO-A activity in the prefrontal cortex and hippocampus. FSF also significantly down-regulated the KLF11, SIRT1 and MAO-A protein expression levels in the prefrontal cortex and hippocampus. These findings indicate that FSF could exhibit an antidepressant-like effect by down-regulating the KLF11/SIRT1-MAO-A pathways, inhibiting MAO-A expression and activity, as well as up-regulating monoamine neurotransmitters levels.

## 1. Introduction

Depression, one of the most common mood disorders, has become one of the major causes of disability and will be the second most significant cause of disease by 2030 [1,2]. Depression is characterized by mood disturbances, cognitive functions, anhedonia and attentional deficits [3]. In the modern world, an estimated 17–21% of the population has suffered from depression [4], and rapid social development will increase the risk factors that cause depression.

Although the pathogenesis of depression is still intangible, some hypotheses have been put forward. For example, the monoamine neurotransmitter hypothesis involves the core concepts of depression [5]. Early studies have shown that lack of monoamine neurotransmitters, such as norepinephrine (NE), 5-hydroxytryptamine (5-HT), dopamine (DA), etc. in the central and peripheral regions is a major cause of depression [6]. Lower concentrations of NE, 5-HT and DA, as well as higher concentrations of 5-hydroxyindoleacetic acid (5-HIAA, 5-HT metabolites), homovanillic acid (HVA, DA metabolites) and 3,4-dihydroxyphenylacetic acid (DOPAC, DA metabolites) were found in the different brain regions with depression. Monoamine oxidases(MAO) enzymes, including two types of MAO terms as MAO-A and MAO-B, could catabolize monoamine neurotransmitters and regulate their levels [5]. Type A monoamine oxidase (MAO-A) catabolizes monoamine neurotransmitters including NE, 5-HT and DA, the abnormally elevated MAO-A activity could result in decreased levels of monoamine transmitters, which in turn leads to depression [7]. Hence, brain MAO-A plays a major role in depressive disorders, could be considered as a target for the treatment of depression [8,9].

The cytostatic protein Kruppel-likefactor11 (KLF11) is a member of the Sp1/KLF zinc finger transcription factor family, its main role is not only to inhibit cell growth and induce apoptosis, but also to act as a MAO transcriptional activator [10]. KLF11 can increase brain MAO-A expression through SP1 binding sites, and play an important role in stress-related depressive disorders [11]. KLF11 protein expression was significantly increased in the frontal cortex and hippocampus, however, no significant changes of KLF11 expression were detected in the striatum and hypothalamus, which suggested that KLF11 protein expression is selectively affected by chronic stress [12]. Silent mating type information regulation 2 homolog 1 (SIRT1) is a protein in the Sirtuin family, and recent studies suggested that SIRT1 protein could affect monoamine transmitter levels in the brain by activating MAO-A transcription, thus the dysregulation of SIRT1 may be involved in depression [13,14].

Stress affects people of all ages, genders and environments, leading to physical and mental health problems. Stress can cause structure degradation and function impairment of the brain, and is an unavoidable phenomenon that is one of the leading risk factors for the development of major depression [15]. The chronic restraint stress (CRS) model is a very common animal model used to mimic the development and progress of clinical depression [16]. Several abnormal behavioral phenotypes such as growth retardation, reduction in sucrose preference and increasing of immobility time are found in the CRS model [17]. Neurotransmitter transmission and metabolic disorder occurred in the prefrontal cortex, hippocampus, striatum, hypothalamus and amygdala in CRS-induced mice [18,19]. 

It has been reported that clorgyline, toloxatone and moclobemide could be used as MAO-A inhibitors, while the side effects, including high cost, a long cycle and low success rate of the drugs noted above could not be ignored. Fortunately, many researchers found that flavonoids, coumarins and alkaloids and their derivatives from natural herbal medicines exhibited strong MAO-A inhibition ability [20]. These natural products became good models for screening MAO-A inhibitors, as they usually show a synergistic manner with antidepressant-like effects, and have almost no side effects [21,22]. A variety of plant extracts and isolated components with MAO-A inhibitory activity are used for depression treatment, and have been extensively studied in many cases, such as *Gynostemma pentaphyllum* [23], *Trigonella foenum-graecum* seeds [24], *Polygala tenuifolia* [25], *Ginkgo biloba* leaves [26], *Astragalus membranaceus* [27], *Radix paeoniae alba* [28] and *Suanzaorenhehuan* Formula [29], etc. In certain cases, attempts to isolate the active monomers from plant extracts eventually may be worth the loss since pharmacological activities often rely on synergistic and polyvalent interactions between plant components [30]. Therefore, in this study we screened the MAO-A inhibitors from seven plant extracts in vitro and determined that the flavonoids from *Trigonella foenum-graecum* seeds (FSF) had the most effective inhibition ability. Then the antidepressant-like effects and mechanisms of FSF in the chronic restraint stress mice model were investigated through behavioral evaluation and neurochemical analysis methods. These data indicate that FSF inhibited the expression and activity of MAO-A by regulating the KLF11/SIRT1-MAO-A signaling pathways and decreased the levels of monoamine neurotransmitters, thereby exhibiting an antidepressant-like effect.

## 2. Results

### 2.1. The Results of Screening for MAO-A Inhibitors in Vitro

**Figure 1:**
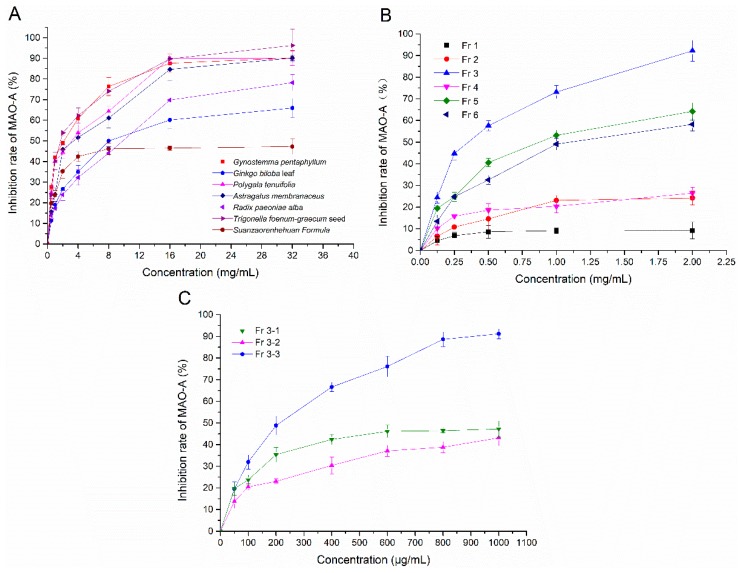
MAO-A inhibition rates of different concentration of samples. (**A**) Plant ethanol extracts. (**B**) Primary fractions by HP-20 macroporous resin from the ethanol extract of *Trigonella foenum-graecum* seeds. (**C**) Secondary fractions by Sephadex LH-20 from Fr 3.

As depicted in Figure 1, with the increase of the concentration of ethanol extracts from 7 plants, the inhibition rate of MAO-A gradually increased in the range of 0.25–32 mg/mL. According to Table 1, the ethanol extract of *Trigonella foenum-graecum* seeds (IC_50_ = 2.968 mg/mL) showed the most significant inhibition MAO-A ability. After purification by HP-20 macroporous resin and Sephadex LH-20, we found that Fr 3-3 (IC_50_ = 0.191 mg/mL) had the most effective MAO-A inhibition ability, but had a lower ability than clorgiline (a selective MAO-A inhibitor, IC_50_ = 0.016 mg/mL).

### 2.2. LC-MS/MS Analysis 

**Figure 2:**
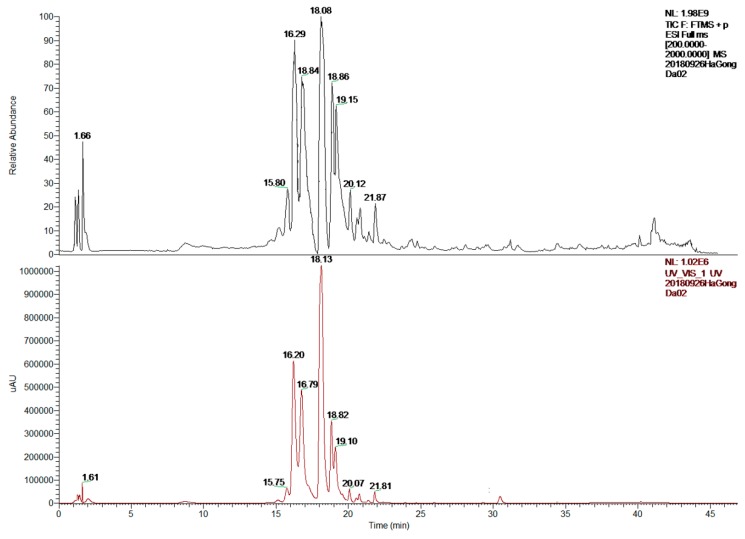
Total ion chromatogram and HPLC chromatogram of Fr3-3 by LC-MS/MS.

Tentative characterization of Fr3-3 was determined by LC-MS/MS analysis in the positive mode. The total ion chromatogram (TIC) of Fr 3-3 was shown in Figure 2. Supported by the fragment pattern (Table 2, Supplementary Materials Figure S1), as well as previous reports, the corresponding compounds were initially determined. Nine representative peaks (Table 2) were identified as Kaempferol 3-(p-coumaryl) glucoside [31], Quercetin 4′-*O*-β-d-glucopyranoside [32], Apigenin 4′,7-*O*-diglucoside [33], Schaftoside [34], Isoschaftoside [35] and Apigenin 8-C-α-d-glucopyranoside [36], respectively, indicating that Fr3-3 with MAO-A inhibition ability includes fenugreek seeds flavonoids (FSF). 

### 2.3. Body Weight

**Figure 3:**
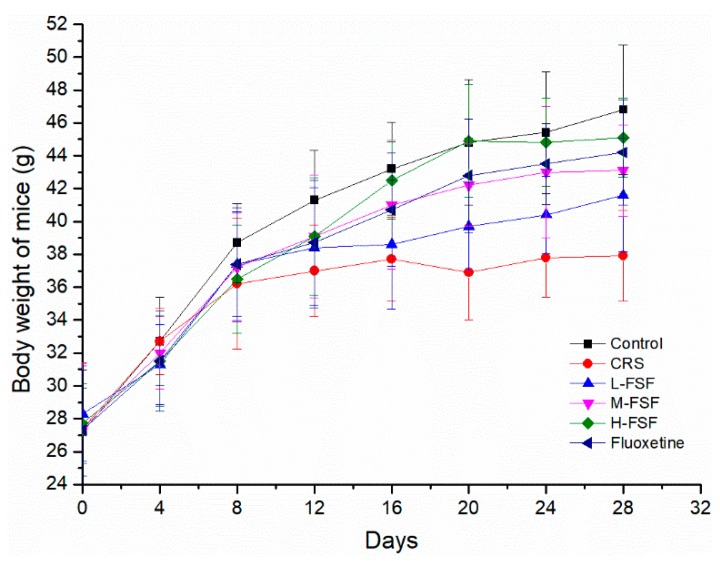
Body weight of mice during the period of chronic restraint stress, n = 10.

Figure 3 sketched these relationships between the body weight of mice and time during the 28 days of chronic restraint stress period. The body weight of the six groups showed an increasing trend with the prolonged feeding time, whereas mice in the CRS group had the slowest weight gain. The body weight in the CRS group was lower than that of other groups on the 8th day of administration. After 28 days of FSF and Flu administration, Flu (10 mg/kg) and different doses of FSF (35, 70 and 140 mg/kg) could effectively alleviate the condition of slow weight gain (*p* < 0.05) compared with the CRS group.

### 2.4. Effects of FSF on Behavioral Tests 

**Figure 4:**
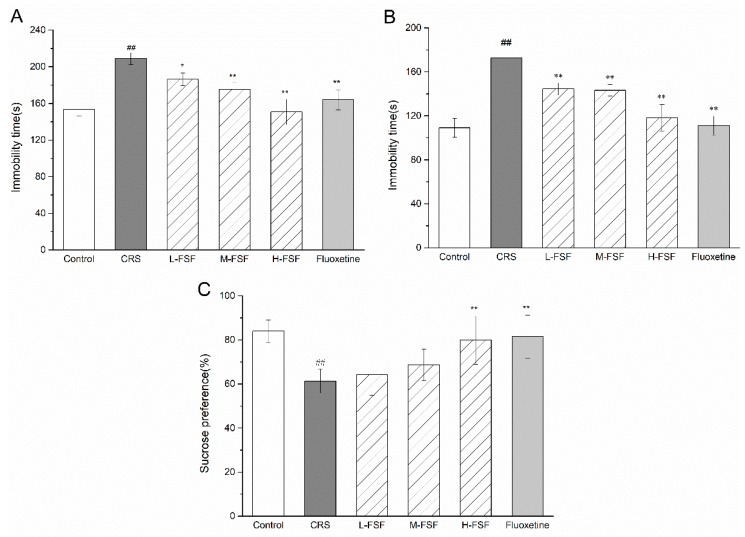
Effects of FSF on depressant-like behaviors in mice. (**A**) Forced swimming test. (**B**) Tail suspension test. (**C**) Sucrose preference test. *n* = 10, ^#^ *p* < 0.05, ^##^ *p* < 0.01 vs. Control group; ^*^ *p* < 0.05, ^**^ *p* < 0.01 vs. CRS treated group.

As depicted in Figure 4, CRS increased immobility time in the forced swimming test and the tail suspension test, produced a depressant-like effect in the animals, and was significantly reduced in FSF and Flu treated mice (*p* < 0.05, *p* < 0.01). After 28 days of the CRS procedure, sucrose preference was significantly reduced (*p* < 0.01). With the treatment of FSF and Flu during CRS, L-FSF (35 mg/kg) and M-FSF (70 mg/kg) treatment did not change the sucrose preference when compared with the CRS group, but H-FSF (140 mg/kg) and Flu (10 mg/kg) treatment significantly increased (*p* < 0.05), indicating that the H-FSF and Flu treatment could ameliorate depression-like behavior (Figure 4C). These results demonstrated the improved effect of FSF on depression-like behavior in CRS mice, similar to fluoxetine.

### 2.5. Effects of FSF on Organ Index

The effect of FSF on organ index in mice is shown in Table 3. Thymus and spleen indices are to be closely bound up in an immune system, and the liver index is related to emotion. In fact, CRS remarkably diminished these indices (*p* < 0.05, *p* < 0.01), whereas M-FSF (70 mg/kg) and H-FSF (140 mg/kg) significantly alleviated the reduction of liver and spleen indices (*p* < 0.05), respectively. FSF showed dose-dependent increase in thymus index. Similarly, the indices of liver, spleen and thymus were markedly restored (*p* < 0.05) after treatment with fluoxetine (10 mg/kg). 

### 2.6. Effects of FSF on Serum CORT Level

**Figure 5:**
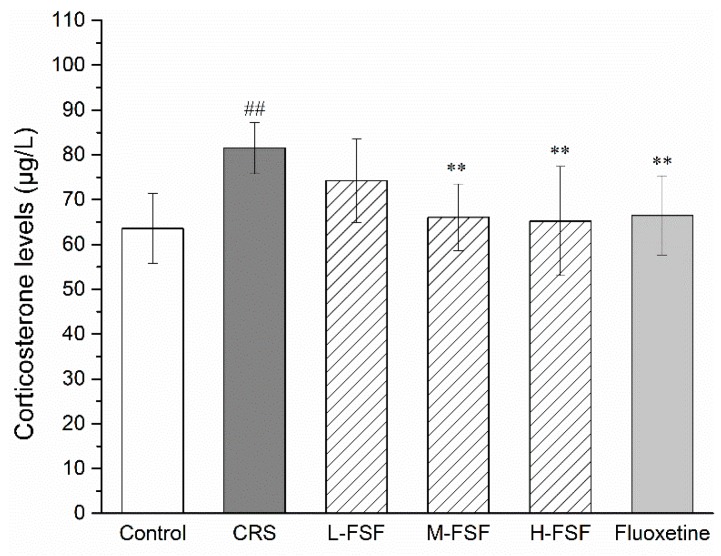
Effects of FSF on serum corticosterone level in mice. *n* = 10, ^##^ *p* < 0.01, vs. Control group; ^**^ *p* < 0.01, vs. CRS group.

The results of serum CORT level are presented in Figure 5. Compared with the control group, the serum CORT level of CRS mice increased significantly (*p* < 0.01), approximately 31%. Significant decreases in serum CORT levels were observed in M-FSF (*p* < 0.01), H-FSF (*p* < 0.01) and Flu group (*p* < 0.05) severally. Unfortunately, there was no significant change in CORT levels in L-FSF. After FSF treatment, there was no significant difference compared with the normal CORT level (*p* > 0.05).

### 2.7. Effects of FSF on NE Level in Different Brain Regions of Mice

**Figure 6:**
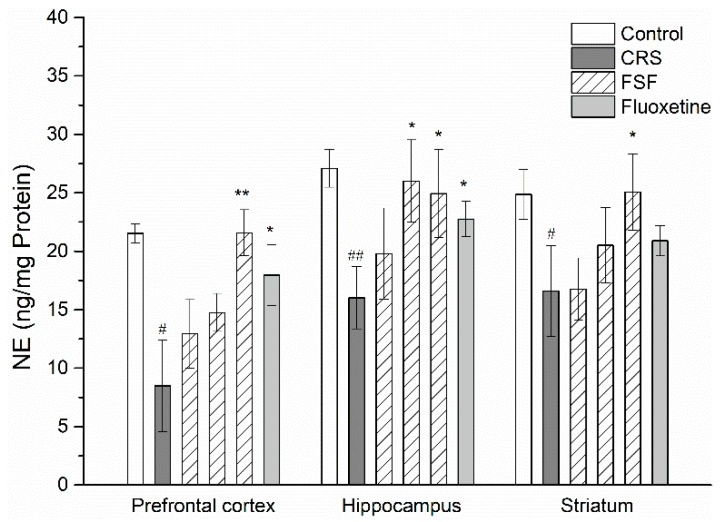
Effects of FSF on NE level in the prefrontal cortex, hippocampus and striatum in mice. *n* = 6, ^#^ *p* < 0.05, ^##^ *p* < 0.01, vs. control group; ^*^ *p* < 0.05, ^**^ *p* < 0.01, vs. CRS group.

The levels of NE detected in the prefrontal cortex, hippocampus and striatum were summarized in Figure 6. Compared with the control group, the CRS group revealed a significant decrease in NE levels in both the prefrontal cortex (*p* < 0.05) and the hippocampus (*p* < 0.01). However, treatment with FSF increased NE levels in the prefrontal cortex, hippocampus and striatum (*p* < 0.05). Flu group also showed a significant increase of NE levels in three brain regions (*p* < 0.05). Additionally, there was no significant diversification in NE levels (*p* > 0.05) between the FSF and control groups.

### 2.8. Effects of FSF on 5-HT and 5-HIAA Levels in Different Brain Regions of Mice

**Figure 7:**
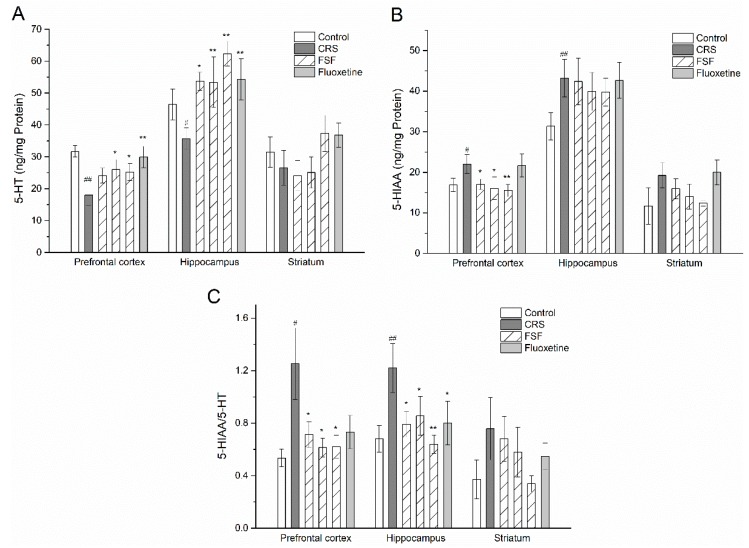
Effects of FSF on 5-HT and its metabolite levels in the prefrontal cortex, hippocampus and striatum in mice. (**A**) The level of 5-HT. (**B**) The level of 5-HIAA. (**C**) The rate of 5-HIAA/5-HT. *n* = 6, ^#^ *p* < 0.05, ^##^ *p* < 0.01, vs. control group; ^*^ *p* < 0.05, ^**^ *p* < 0.01, vs. CRS group.

Effects of CRS-induced stress on 5-HT and its metabolite (5-HIAA) levels in mice were depicted in Figure 7. The CRS induced a significant decrease in 5-HT levels (*p* < 0.05) and a significant increase in 5-HIAA levels (*p* < 0.05) in the both prefrontal cortex and hippocampus but not striatum, compared with the control group. Pretreatment with FSF reversed the decreased 5-HT levels induced by CRS (*p* < 0.05). The turnover rates of 5-HT dramatically increased (*p* < 0.05) in the prefrontal cortex and hippocampus. However, Flu did not have the same effect on the turnover rate of 5-HT (Figure 7C).

### 2.9. Effects of FSF on DA, DOPAC, and HVA Levels in Different Brain Regions of Mice

**Figure 8:**
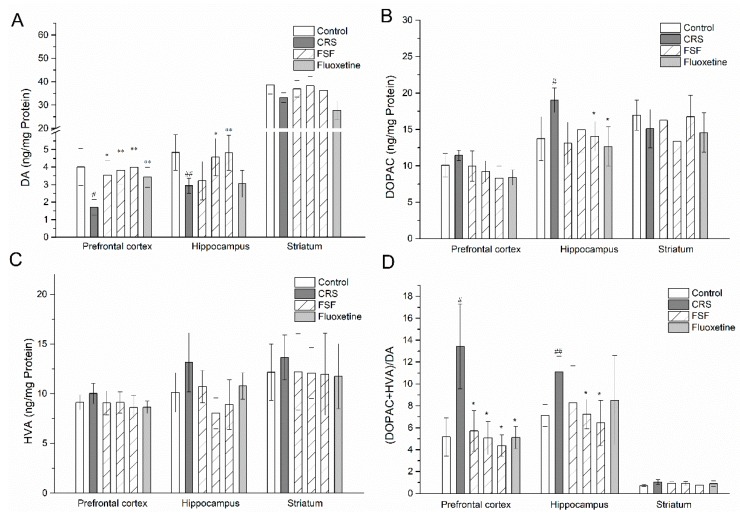
Effects of FSF on DA and its metabolites levels in the prefrontal cortex, hippocampus and striatum in mice. (**A**) The level of DA. (**B**) The level of DOPAC. (**C**) The level of HVA. (**D**) The rate of (DOPAC+HVA)/DA. *n* = 6, ^#^ *p* < 0.05, ^##^ *p* < 0.01, vs. Control group; ^*^ *p* < 0.05, ^**^ *p* < 0.01, vs. CRS group.

The effects of FSF and Flu on the DA and its metabolite (DOPAC and HVA) levels in the brains of mice exposed to the CRS were shown in Figure 8. After a period of four weeks stress administration, DA levels were drastically reduced in the prefrontal cortex (*p* < 0.05) and hippocampus (*p* < 0.01). Compared with the CRS group, FSF dose-dependently increased DA levels in the prefrontal cortex (*p* < 0.05, *p* < 0.01); M-FSF (70 mg/kg), H-FSF (140 mg/kg) and Flu (10 mg/kg) showed an increase (*p* < 0.01) in DA in the hippocampus; but the DA levels did not show any significant effects in the striatum. Meanwhile, H-FSF and Flu could reverse the increase of DOPAC levels in the hippocampus induced by CRS. However, DOPAC levels showed no statistical difference in either the prefrontal cortex or the striatum. There was no significant difference in the HVA levels in the three brain regions. In addition, the turnover rate of DA in the prefrontal cortex and hippocampus in the CRS group increased (*p* < 0.05) compared to that in the control group (Figure 8D). However, supplementation of FSF contributed to decrease the turnover rates of DA in both the prefrontal cortex and hippocampus.

### 2.10. Effects of FSF on MAO-A Activity in Different Brain Regions of Mice

**Figure 9:**
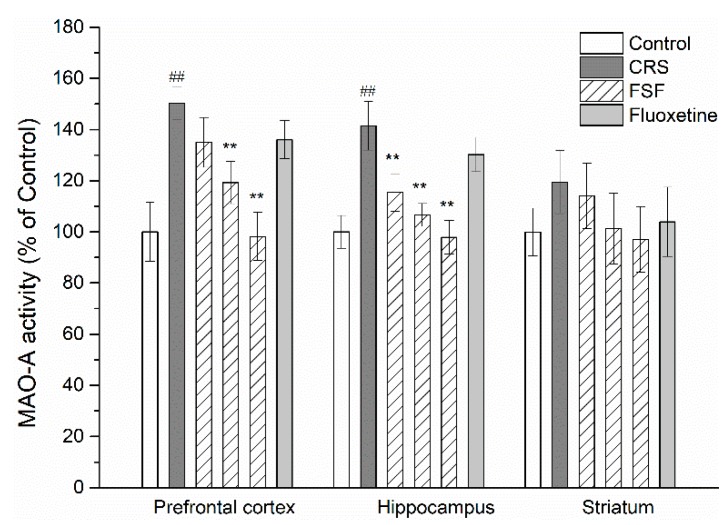
Effects of FSF on enzyme activities in the prefrontal cortex, hippocampus and striatum in mice. *n* = 6, ^##^ *p* < 0.01, vs. control group; ^**^ *p* < 0.01, vs. CRS group.

The activities of MAO-A detected in the prefrontal cortex, hippocampus and striatum were summarized in Figure 9. The MAO-A activity in the prefrontal cortex and hippocampus in CRS mice was increased significantly by about 1.5-fold (*p* < 0.01) and 1.4-fold (*p* < 0.01), compared with the control group. The MAO-A activity in the prefrontal cortex and hippocampus was significantly reduced by the supplementation of FSF in a dose dependent manner (*p* < 0.01). Meanwhile, with FSF treatment, the reduction of MAO-A activity is controllable, reducing to normal levels. In addition, fluoxetine had no meaningful effect on MAO-A activity in three brain regions. 

### 2.11. Effects of FSF on KLF11, SIRT1 and MAO-A Protein Level in Different Brain Regions of Mice

**Figure 10:**
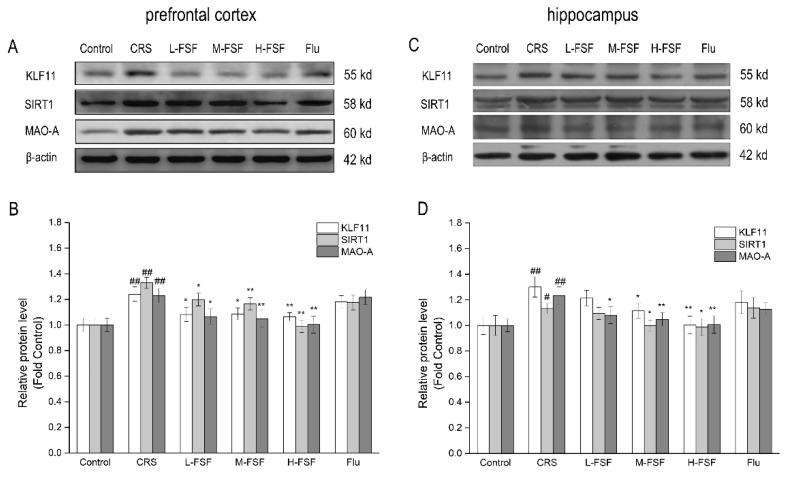
Effects of FSF on KLF11, SIRT1 and MAO-A protein level in the prefrontal cortex and hippocampus in mice. (**A**) Representative bands of protein in the prefrontal cortex. (**B**) The relative protein level in the prefrontal cortex. (**C**) Representative bands of protein in the hippocampus. (**D**) The relative protein level in the hippocampus. ^#^ *p* < 0.05, ^##^ *p* < 0.01, vs. control group; ^*^ *p* < 0.05, ^**^ *p* < 0.01, vs. CRS group.

The protein levels of KLF11, SIRT1 and MAO-A determined by western blot analysis were shown in Figure 10. In the prefrontal cortex, the results showed a significant increase (*p* < 0.01) in KLF11, SIRT1 and MAO-A protein expression between the Control and CRS groups. Meanwhile, there are similar results in the hippocampus. Compared with the CRS group, the protein levels decreased significantly (*p* < 0.05, *p* < 0.01) after the FSF treatment in the prefrontal cortex and hippocampus, and there was a dependence on the FSF dose. However, there was no significant effect found with fluoxetine treatment on KLF11, SIRT1 and MAO-A protein expression in the prefrontal cortex and hippocampus.

## 3. Discussion

Stress can interfere with the nervous system, which is positively associated with the onset of depression [37,38]. Chronic restraint stress could produce persistent depressive-like symptoms in mice, which has been put forward as a widely used animal model of depression. The brain is the main target of stress-related psychiatric disorders [39,40]. In the present study, our studies establish that FSF exhibited the most effective MAO-A inhibition ability. Furthermore, the results demonstrate the KLF11/SIRT1-MAO-A signaling pathways and reveal the antidepressant-like effect of FSF mainly via modulation of monoamine regulatory pathways.

The decreased body weight after 28 days CRS exposure was shown in the present study. To a certain extent, weight loss could be used to assess the occurrence of depression. The extension of the immobility time in FST and TST can effectively reflect the behavioral despair and hopelessness, and sucrose preference is a behavioral indicator reflecting the degree of pleasure loss. In this study, we observed increased immobility in FST and TST as well as reduced sucrose preference after the CRS protocol, indicating that depression model was built successfully. Recent studies have also noted that CRS can induce depression-like behaviors in mice [41], which is in agreement with our results. The increased serum CORT level in CRS mice indicated that CRS could make the HPA axis hyperactive, and then lead to an imbalance of hormone levels in the body, particularly in the brain. However, treating mice with FSF and fluoxetine before the stressful events reversed the harmful change (reduced body weight and sucrose preference, increased immobility time and serum CORT levels in mice).

Previous studies have indicated that chronic stress could reduce monoamine transmitters (NE, 5-HT and DA) levels in the brain and induce depressive symptoms [42,43]. Since prefrontal cortex, hippocampus and striatum are associated with important functions such as mood, motivation, behavioral control, learning and memory [44], we chose these three brain regions for research. FSF has been proven to alleviate depressive-like symptoms and to increase the NE level in three brain regions. The levels of 5-HT and DA in the prefrontal cortex and hippocampus increased dramatically after FSF treatment, compared with the CRS group. Additionally, there was no significant diversification (*p* > 0.05) from the normal monoamine transmitters levels in the brain after FSF treatment. We detected that the turnover rate of 5-HT and DA decreased notably in the prefrontal cortex and hippocampus, which indicated a reduction in 5-HT and DA metabolism associated with MAO-A activity.

MAO-A participates in the metabolism of 5-HT, NE and DA, and brain MAO-A activity is positively correlated with depression [5]. To further address the molecular mechanism in regulation of neurotransmitter levels after FSF treatment, we measured the MAO-A activity in the three brain regions. We discovered that MAO-A activity was overactivated in the prefrontal cortex and hippocampus in the CRS group compared with the control group. However, there was no significant change in the striatum. With the treatment of FSF, the MAO-A activity was inhibited. However, fluoxetine had no effect on MAO-A activity. Results from several studies have identified that the severity of depressive symptoms is associated with reduced MAO-A activity and increased neurotransmitter levels [5,9,45].

KLF11 and SIRT1 are the key transcriptional up-regulators of MAO-A. KLF11 directly regulates MAO-A gene transcription, and SIRT1 indirectly regulates MAO-A gene transcription through deacetylase and activation of NHLH2 and FOXO1 [11,46]. Our studies showed that the CRS group displayed an increase in protein expression of KLF11, SIRT1 and MAO-A compared with the control group, indicating that the KLF11-MAO-A and SIRT1-MAO-A pathways were overactivated by CRS exposure. FSF could inhibit MAO-A activity and reduce the expression of KLF11 and SIRT1 proteins, thereby decrease MAO-A protein level. The result is consistent with the report that MAO-A inhibitors treatment during chronic stress exposure downregulates the KLF11-MAO-A and SIRT1-MAO-A pathways [46,47]. However, fluoxetine has no significant effect on KLF11, SIRT1 and MAO-A protein expression in the prefrontal cortex and hippocampus, which suggested that fluoxetine did not participate in this pathway for antidepressant effects. Fluoxetine selectively inhibits 5-HT transporters, blocks pre-synaptic membrane reuptake of 5-HT, prolongs and increases 5-HT action, resulting in antidepressant effects.

## 4. Materials and Methods

### 4.1. Chemicals and Drugs

*Gynostemma pentaphyllum*, *Trigonella foenum-graecum* seeds, *Polygala tenuifolia*, *Ginkgo biloba* leaves, *Astragalus membranaceus*, *Radix paeoniae alba* and *Suanzaorenhehuan* were purchased from Harbin Xinheng Traditional Chinese Medicine Materials Ltd. (Harbin, China) and authenticated by Professor Lianjie Su, Heilongjiang University of Chinese Medicine. HP-20 macroporous resin and Sephadex LH-20 were obtained from Soledad Technology Co., Ltd. (Beijing, China). Clorgiline, pargilin, fluoxetine (Flu) were purchased from Sigma (St. Louis, MO, USA). Casein, vanilla acid, 4-aminoantipyrine and horseradish peroxidase were purchased from Aladdin Bio-Chem Technology Co., Ltd. (Shanghai, China). 

### 4.2. Extraction and Isolation

All plants were extracted with 60% (*v/v*) ethanol, under sonication for 60 min at 50 °C for two times and filtered and evaporated at 45 °C. Then MAO-A inhibition activity of each extract was evaluated, and the ethanol extract of *Trigonella foenum-graecum* seeds showed the most significant inhibition. The extract was separated and purified by HP-20 macroporous resin eluted with water, 10%, 30%, 50%, 70%, 90% ethanol. The eluted fractions (Fr 1, Fr 2, Fr 3, Fr 4, Fr 5, Fr 6) were concentrated and dried. The MAO-A inhibition activity was investigated, and the Fr 3 showed the most significant inhibition. To further identify Fr 3, we adopted Sephadex LH-20 to analyze the active fractions in Fr 3 by using methanol and water (30:70), and obtained three fractions (Fr 3-1, Fr 3-2, Fr 3-3). Each fraction was evaluated for MAO-A inhibition again, and the results showed that the Fr 3-3 exhibit the most significant inhibition. Then Fr 3-3 was obtained and stored at 4 °C for the next experiments.

### 4.3. Screening for MAO-A Inhibitors In Vitro

Extraction and preparation of MAO refer to previous methods [48]. The crude enzyme solution was diluted 4 times, and the corresponding volume of 500 nmol/L pargilin solution (a selective MAO-B inhibitor) was pre-incubated for 30 min to completely inhibit MAO-B, and the MAO-A working solution was prepared [49]. Then, 40 μL enzyme and 40 μL sample solution of different concentrations were added to the 96-well plate. After incubation at 37 °C, add 120 μL substrate was immediately added (2.5 mmol/L casein, prepared from 0.2 mol/L PH 7.6 PBS), as was 40 μL developing solution (1 mmol/L vanilla acid, 0.5 mmol/L 4-aminoantipyrine, 4 U/mL horseradish peroxidase, by 0.2 mol/L PH 7. 6 of PBS). After incubation at 37 °C, the absorbance value of 490 nm was determined by using a multifunctional enzyme marker (Varioskan Flash, Thermo Fisher Scientific, Waltham, MA, USA).

### 4.4. LC-MS/MS Analysis

The Fr3-3 was analyzed by using LC-MS/MS (APEX IV, FT-MS, Bruker, USA) equipped with Extend-C18 column (1.8 µm, 2.1 × 50 mm) with UV detection at 280 nm. Acetonitrile (A) and acidified water (B, 0.1% formic acid, *v/v*) were elution solvent. Elution program: 0–20 min (5–25% A), 20–30 min (25–40% A), 30–40 min (40–70% A), 40–60 min (70–100% A). The column temperature was 30 °C, the flow rate was 0.5 mL/min, and the sample loading was 10 μL. Major parameters: spray voltage, 3000 V; evaporator temperature, 300 °C; sheath gas (N_2_) pressure, 4.0 bar; and scan range, *m/z* 150–1000. The collision energy was initially set at 10 eV for the MS/MS experiments of the preferred ions and then modified according to the fragments.

### 4.5. Animals and Experimental Design

Male KM mice (18–22 g) were purchased from the Animal Center of Harbin Medical University (SCXK(Hei)2006-008). All experimental procedures were approved by the Animal Research Committee of Harbin Medical University. The animals were housed in cages (23 ± 2 °C, 12-h light and 12-h dark periods) with *ad libitum* access to food and water. After 7 days of adaptive feeding, animals were randomly separated into the following 6 experimental groups (10 mice per group): Control group, Chronic Restraint Stress group (CRS), Fluoxetine group (Flu, 10 mg/kg, p.o., as positive group), L-FSF group (35 mg/kg, p.o.), M-FSF (70 mg/kg, p.o.) and H-FSF (140 mg/kg, p.o.). Control group was given equal volume of distilled water. In addition to the control group, the remaining five groups were exposed to CRS for 28 days. 

### 4.6. Chronic Restraint Stress Protocol

The CRS was performed by adapting the procedure used by Shuichi et al. [50]. Restraint stress was applied 4 h/d between 9 A.M. and 1 P.M. for 28 d with acrylic cylinder (inner diameter 6.5 cm, length 20.0 cm, vent hole at the end of the cylinder) that can effectively immobilize the mice. After 4 h of restraint stress, the mice *ad libitum* accessed food and water. 

### 4.7. Behavioral Tests

#### 4.7.1. Forced Swimming Test

A forced swimming test was performed with some previously reported methods and several modifications were made [51]. The mice were placed in a glass (25 cm high × 15 cm diameter) containing 25 cm of water (25 ± 1 °C). On the first day, the mice were placed in a glass for 15 min to induce a helpless state, and then they were returned to the glass for 6 min on the second day, and the immobility time was finally measured over 4 min. Immobility was defined as the action required to keep the heads of all the mice above the water surface without all movements.

#### 4.7.2. Tail Suspension Test

Tail suspension test was implemented according to a previously reported method [52]. The mice tails were fixed on the top of the soundproof box with medical tape, and the heads were 20 cm from the floor. The mice were suspended for 6 min and the immobility time was measured at last 4 min. Immobility time was defined as the lack of escape-oriented behavior. Moreover, when mice did not have any physical movement, they were considered to be passively hanging and not moving at all.

#### 4.7.3. Sucrose Preference Test

Sucrose preference test was performed according to a previously described method [53], with some modifications. Twenty-four hours prior to testing, mice were not allowed to drink water. After that the mice were given two bottles of water and 1% sucrose solution at the same time. In order to determine the sucrose preference in mice, the weight of each bottle was weighed and the amount of solution consumed was calculated after 24 h. 

### 4.8. Blood and Tissue Sample Collection

The blood samples were collected by removing eyeball and kept at room temperature for 1 h, centrifuged at 3000 rpm for 20 min to obtain serum, and stored at −80 °C for the subsequent corticosterone assays. The mice were decapitated and the brains were quickly removed on ice. The prefrontal cortex, hippocampus and striatum were isolated according to the reported protocol [54], and were stored at −80 °C. The prefrontal cortex, hippocampus and striatum were homogenized with cold phosphate buffer (pH 7.4, 0.05 M, 1:10 *w/v*) using a Teflon-glass homogenizer. 

### 4.9. Determination of CORT, Monoamines and Its Metabolites

The levels of serum CORT and monoamine neurotransmitters (NE, 5-HT, 5-HIAA, DA, DOPAC and HVA) were measured using commercial ELISA kits (Mercker Biological Technology Co., Ltd., Wuhan, China) according to the manufacturer’s instructions. The absorbance was measured at 450 nm using a microplate reader (Multiskan Spectrum, Thermo Fisher Scientific, USA) within 15 min. Protein concentrations were estimated by the BCA method using a BSA protein analysis kit (Nanjing Bioengineering Institute, Nanjing, China).

### 4.10. Measurements of MAO-A Activity

The MAO-A activity was performed using a previously reported method [9], with some modification. Brain tissue homogenates were then centrifuged at 3000 rpm for 5 min at 4 °C twice. The supernatant was transferred to a new tube and centrifuged at 12,000 rpm for 10 min at 4 °C. The pellets were resuspended in the same buffer. The MAO-A activity was measured using a MAO-Glo^TM^ kit (Promega, WI, USA) according to the manufacturer’s instructions. The data were measured by using a multifunctional enzyme marker (Varioskan Flash, Thermo Fisher Scientific, USA). Protein concentrations were estimated by the BCA method using a BSA protein analysis kit (Nanjing Jiancheng Bioengineering Institute, Nanjing, China).

### 4.11. Western Blot Analysis

The prefrontal cortex and hippocampus were homogenized in a RIPA buffer. Lysates were centrifuged at 4 °C, 12,000 rpm for 10 min, and the supernatant was obtained and stored at −20 °C. Protein concentrations were estimated by the BCA method using BSA protein analysis kit. Twenty micrograms of total protein from each sample were separated in 10.5% SDS-PAGE gel by electrophoresis and transferred onto a PVDF membrane. The membrane was blocked with 5% non-fat dry milk for 1 h at room temperature and incubated with rabbit anti-MAO-A antibody, rabbit anti-KLF11 antibody and rabbit anti-SIRT1 antibody (1:1000; bs-6679R, bs-16096R, bs-0921R; Biosynthesis Biotechnology, China) for 1 h at room temperature. Then, the membrane was incubated with horseradish peroxidase-conjugated secondary antibodies for 2 h at room temperature to visualize blots. β-Actin was used as an internal reference protein. 

### 4.12. Data Analysis

Data were expressed as mean ± SEM and analyzed by one-way ANOVA, and then Tukey test and Dunnet’s multiple comparisons were performed using SPSS version 16.0 statistical software (IBM, New York, NY, USA). Differences in *p* < 0.05 were considered significant in all tests.

## 5. Conclusions

In summary, the present study revealed the antidepressant effect of flavonoids from *Trigonella Foenum-Graecum* seeds that could inhibit the expression and activity of MAO-A by down-regulating the KLF11-MAO-A and SIRT1-MAO-A signal pathways, and by regulating the levels of monoamine neurotransmitters (NE, 5-HT, DA and their metabolites) in the prefrontal cortex, hippocampus and striatum in chronic restraint stress mice. Further research in animal studies is needed to better understand the potential impacts of these findings. 

## Figures and Tables

**Table 1:** IC_50_ values of MAO-A inhibition rates.

Ethanol Extracts/Purified Fractions	MAO-A Inhibition IC50 (mg/mL)
*Gynostemma pentaphyllum*	3.322
*Ginkgo biloba* leaves	16.445
*Polygala tenuifolia*	4.470
*Trigonella foenum-graecum* seeds	2.968
*Astragalus membranaceus*	7.682
*Radix paeoniae alba*	12.568
*Suanzaorenhehuan Formula*	27.827
Fr 1	21.022
Fr 2	7.152
Fr 3	0.947
Fr 4	8.240
Fr 5	2.377
Fr 6	2.862
Fr 3-1	0.985
Fr 3-2	1.878
Fr 3-3	0.191
Clorgiline	0.016

**Table 2:** Mass spectral data positive mode.

Peak	RT (min)	MW	[M + H]^+^ (*m/z*)	MS^2^ (*m/z*)	Molecular Formula	Identification
1	15.80	594	595	431, 147	C_30_H_26_O_13_	Kaempferol 3-(p-coumaryl) glucoside
2	16.29	464	465	303	C_21_H_20_O_12_	Quercetin 4′-*O*-β-d-glucopyranoside
3	16.84	594	595	433, 271	C_27_H_30_O_15_	Apigenin 4′,7-*O*-diglucoside
4	18.08	564	565	529, 499, 391	C_26_H_28_O_14_	Schaftoside
5	18.86	564	565	547, 511, 427	C_26_H_28_O_14_	Isoschaftoside
6	21.87	432	433	415, 397, 313, 283	C_21_H_20_O_10_	Apigenin 8-C-α-d-glucopyranoside

**Table 3:** Effects of FSF on the organ index in mice.

Group	Liver Index (mg/g)	Spleen Index (mg/g)	Thymus Index (mg/g)
Control	56.33 ± 4.39	2.70 ± 0.63	2.66 ± 0.82
CRS	50.84 ± 2.48 ^#^	2.38 ± 0.37 ^#^	2.07 ± 0.76 ^##^
L-FSF	51.51 ± 4.32	2.50 ± 0.25	2.41 ± 0.85 ^*^
M-FSF	56.33 ± 4.39 ^*^	2.79 ± 0.37 ^*^	2.40 ± 0.79 ^*^
H-FSF	56.73 ± 6.40 ^*^	2.79 ± 0.25 ^*^	2.66 ± 0.82 ^**^
Fluoxetine	56.27 ± 7.33 ^*^	2.87 ± 0.61 ^*^	2.50 ± 0.73 ^*^

*n* = 10, ^#^ *p* < 0.05, ^##^ *p* < 0.01, vs. Control group; ^*^ *p* < 0.05, ^**^ *p* < 0.01, vs. CRS group.

## Supplementary Materials

The following are available online. Figure S1: The mass spectra and chemical structures of compounds of FSF.
