# Supplementary material for: The Antidepressant-like Effect of Flavonoids from Trigonella Foenum-Graecum Seeds in Chronic Restraint Stress Mice via Modulation of Monoamine Regulatory Pathways
Source: Molecules. 2019 Mar 20;24(6):1105. doi: 10.3390/molecules24061105 (PMC6471463; doi:10.3390/molecules24061105)
Supplement: Supplementary file 1 [file molecules-24-01105-s001.pdf]

RT=15.80 min

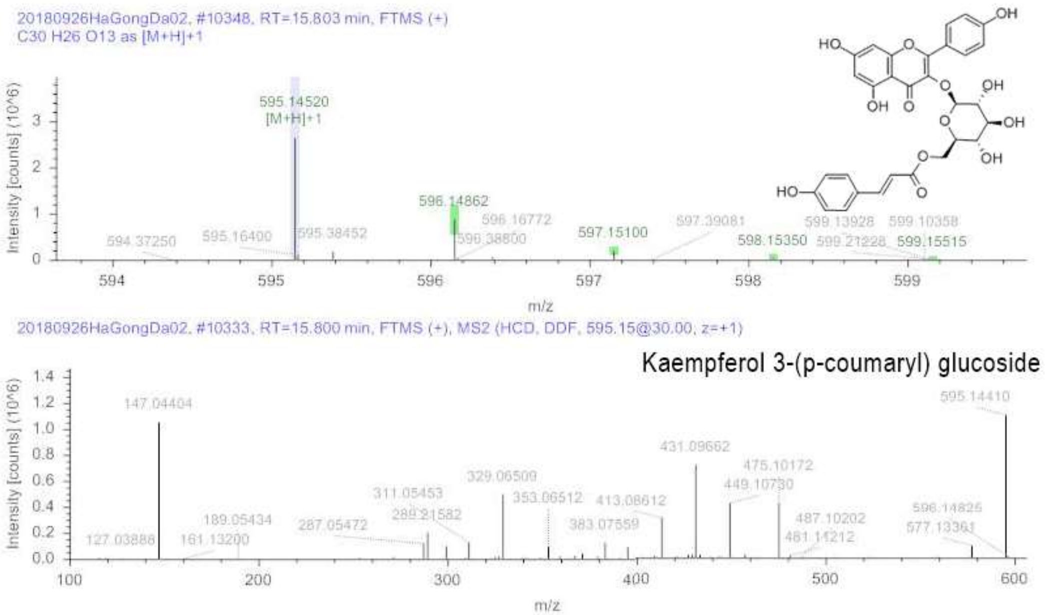

RT=16.29 min

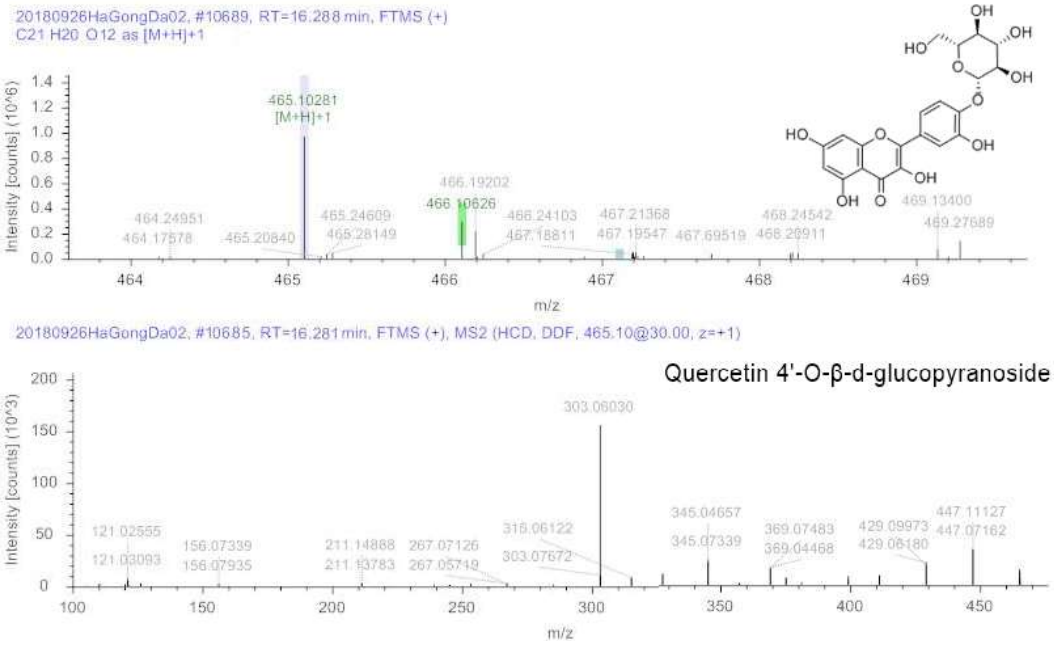

RT=16.84 min

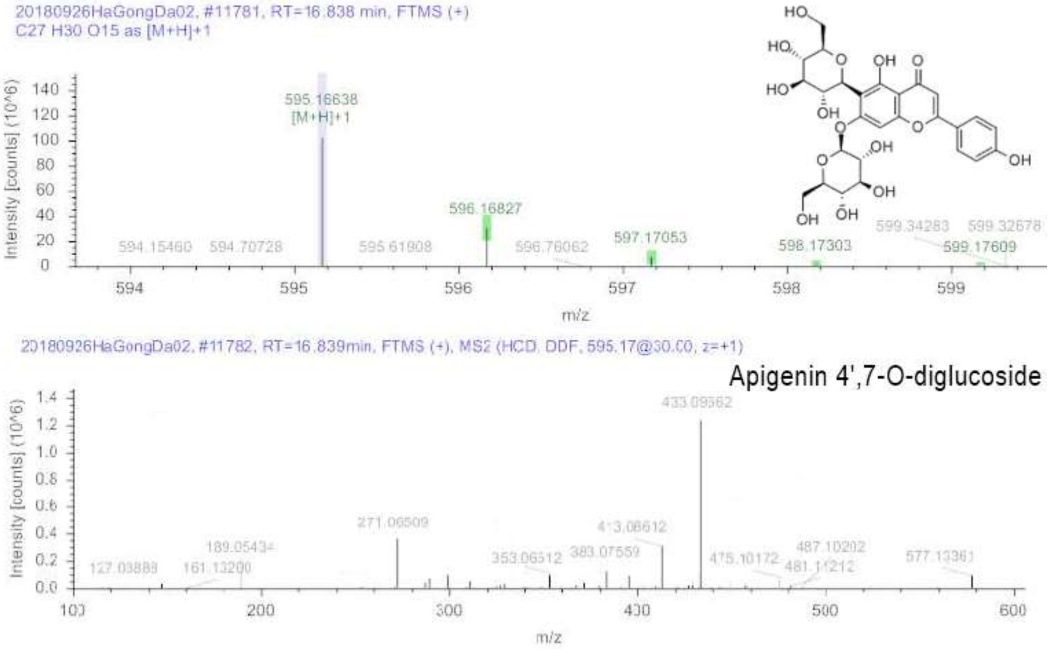

RT=18.08 min

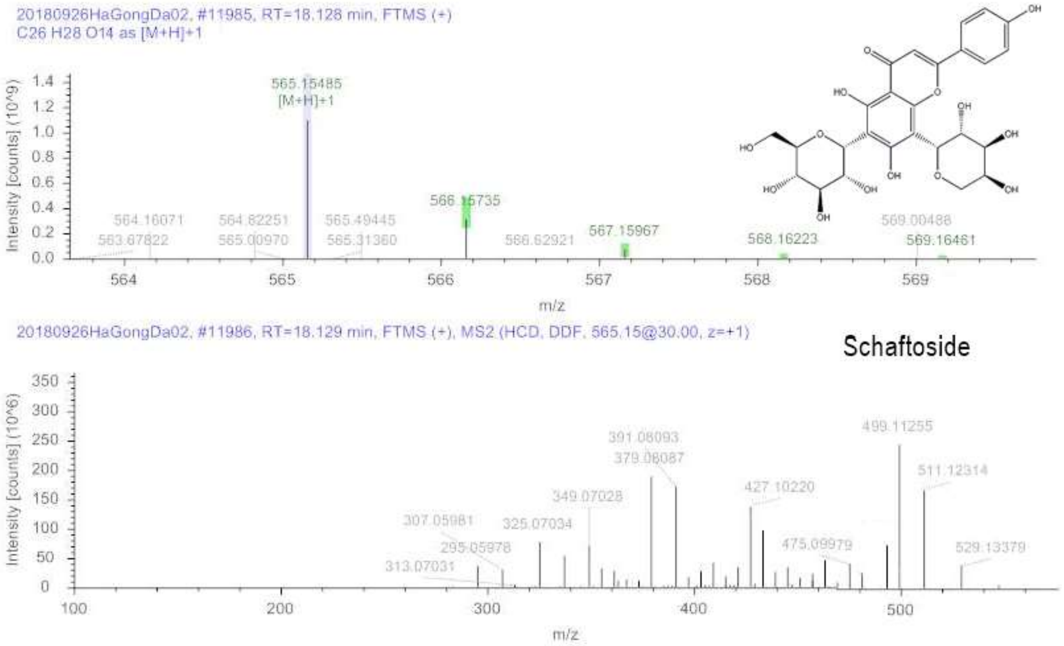

RT=18.86 min

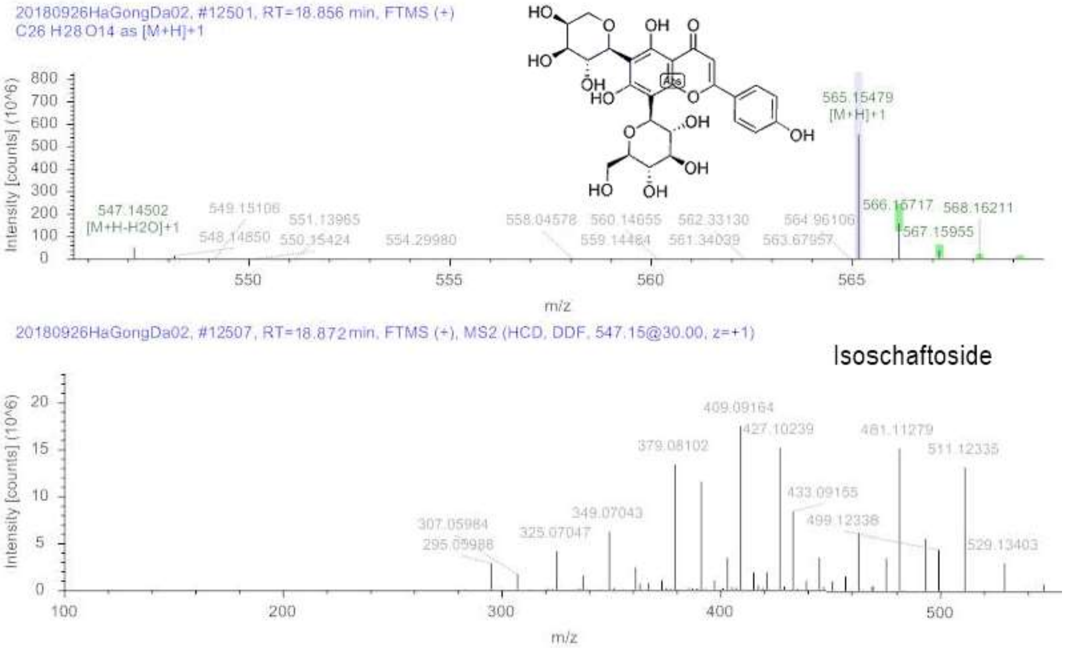

RT=21.87 min

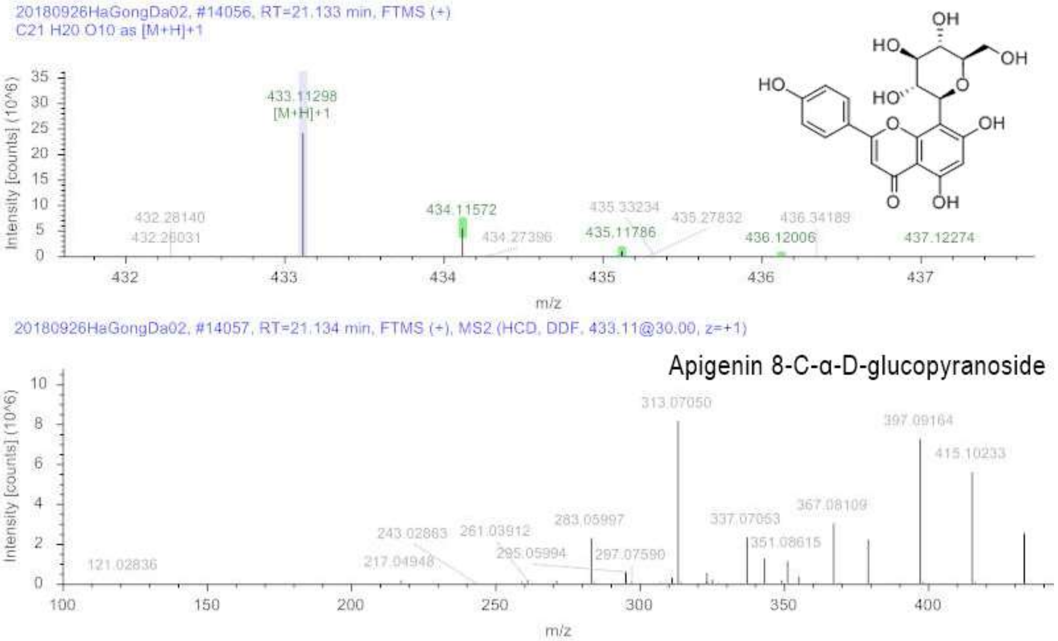

Figure S1. The mass spectra and chemical structures of compounds of FSF.
